# Night shift work, short sleep and obesity

**DOI:** 10.1186/s13098-020-0524-9

**Published:** 2020-02-10

**Authors:** Maria Carlota Borba Brum, Fábio Fernandes Dantas Filho, Claúdia Carolina Schnorr, Otávio Azevedo Bertoletti, Gustavo Borchardt Bottega, Ticiana da Costa Rodrigues

**Affiliations:** 1 Division of Occupational Medicine, Hospital de Clinicas de Porto Alegre, Ramiro Barcelos, Porto Alegre, Rio Grande do Sul 2350 Brazil; 2 Department of Internal Medicine, Universidade Federal do Rio Grande do Sul-Porto Alegre, Ramiro Barcelos, Porto Alegre, Rio Grande do Sul 2350 Brazil; 3 Division of Endocrinology, Hospital de Clinicas de Porto Alegre, Ramiro Barcelos, Porto Alegre, Rio Grande do Sul 2350 Brazil

**Keywords:** Night work, Abdominal obesity, Social jetlag, Health care workers, Shift work

## Abstract

**Background:**

Obesity is associated with increased general mortality and comorbidities, it is multifactorial and some evidence has shown that sleep duration and shift work may be implicated in its pathogenesis.

**Objectives:**

The aim of this study was to evaluate the association between shift work, quality of life and obesity among healthcare workers of a Brazilian University Hospital.

**Methods:**

A cross-sectional study was performed from April 2013 to December 2014 with 200 workers of a University Hospital. Sociodemographic data were evaluated and BREF WHOQOL was used for quality of life. The physical activity was evaluated using the International Physical Activity Questionnaire (IPAQ), Chronotypes and daily sleep preference were investigated using Munich Chronotype Questionnaire (MCTQ). Venous blood was collected after 12-h of fasting for laboratory tests.

**Results:**

In this sample, the night shift workers had higher income and were older compared to day shift workers. Night shift workers sleep less hours, had higher weight, body mass index and abdominal circumference when compared to the day shift workers. Night shift workers had almost 3 times higher association with abdominal obesity independent of age and gender, than day shift workers. MCTQ parameters showed that night shift workers had lower sleep duration during working days and also during free days, associated with a higher level of social jetlag. Social jetlag had an association with obesity. We found no difference for quality of life between shifts.

**Conclusions:**

Night work was a risk factor for abdominal obesity, social jetlag was higher in night shift workers and it was associated with presence of obesity.

## Background

Shift work has been definitively incorporated into society, and is no longer limited to essential services such as health, public safety and heavy industry, but also occurs in other goods and services production branches. Shift work can be continuous, where workers follow each other at the workplace, or in rotating shifts [1]. In the USA and Europe it is estimated that about 18 to 20% of workers are involved in night work or in shifts [2].

Night work or shift work presents common characteristics which may interfere in the circadian physiological process causing desynchronization. Exposure to continuous light during the night, frequent snacks, little physical activity, nocturnal eating habits and nocturnal physical activities are among the possible triggering factors [3].

Several studies have demonstrated that shift work may induce the development of dyslipidemias, diabetes mellitus [4, 5] and systemic arterial hypertension (SAH). The prevalence of obesity has grown gradually over the last few decades in the USA [6] and is associated with an increase in overall mortality and comorbidities secondary to the disease [7]. It is acknowledged that life style related factors, such as the excessive intake of calories and sedentary life are the main factors responsible for increased obesity. However, other factors, besides genetic profile, have been implicated in its pathogenesis, such as duration of sleep and shift work. Some authors observed a positive relationship between night work or shifts with the increased body mass index (BMI), and this is significantly greater compared to the day shift workers [8, 9]. Knutson et al. found that the reduction of sleep duration may also increase the risk of cardiovascular diseases, insulin resistance, diabetes as well as obesity [10].

Short sleep (defined as ≤ 6 h of sleep/day) [11] and sleep disorders due to shift work, have been associated with negative effects on the hormone profile and a positive energy balance [12]. Restricted sleep has metabolic and endocrine consequences, including reduction of glucose tolerance and insulin sensitivity, increased serum concentration of nocturnal cortisol, rise of ghrelin levels, reduction of leptin levels and an increased feeling of hunger and appetite [13–15]. In this sense, it is believed that the reduction in the number of hours of sleep may be related to the increased incidence of obesity and overweight, although the mechanisms of such a relationship are not yet fully known [7].

The objective of this study was to evaluate the association between night work and obesity, as well as quality of life among the workers in the South of Brazil.

## Materials and methods

The study was performed during the period from April 2013 to December 2014; participants were recruited during annual medical exams at a university hospital in the city of Porto Alegre, southern Brazil. The study included 200 employees from the departments of administration, support, direct patient care, and maintenance of hospital machinery and equipment, who worked in the day and night shifts and accepted to participate in the study. The exclusion criteria for the study included temporary employees, those who were off work for more than 15 days, pregnant women or employees who were in the process of retiring.

Regarding the working hours, the day shift was from 6 to 8 h a day (from 7 A.M. to 1 P.M., from 8 A.M. to 5 P.M. and from 1 P.M. to 7 P.M.), on work scales of 5 days/2 days, 6 days/1 day, considering days of work and days of rest, and on duty periods of 12 h on weekends. For the night shift, the working hours were from 6 to 12 h (from 6 PM to midnight and from 7 PM to 7 AM) on work scales of 12/60 h or 12/24 h, or 6 nights/1 day, 5 nights/2 days, or 4 nights/3 days, considering the days on duty and the days of rest.

The information concerning age (collected as a continuous variable), race (classified as white or other), gender, marital status (classified as with or without a partner), number of children, level of schooling (categorized as years of study), professional activity (categorized as administrative, health professionals, support and maintenance), time in the company, working hours, time on shift, work scales, another job, shift at another job, personal history of hypertension, diabetes, angina, dyslipidemia and glucose intolerance, smoking habits (self-reported as a smoker or non-smoker last month), drinking alcoholic beverages (self-reported as to which drink, consumption of time and number of servings per week). In order to evaluate the health status, a physical examination was performed (weight, height, blood pressure, heart rate, measurements of abdominal circumference) and venous blood collected after 12 h of fasting for laboratory tests.

Glycated hemoglobin levels were measured by high-performance liquid chromatography (Variant II turbo^®^) (reference range 4.7–6.0%), insulin was measured by the microparticle chemiluminescent immunoassay method (Architect Ci4100^®^). Fasting plasma glucose was measured by the enzymatic UV hexokinase method (Cobas c702^®^). Serum creatinine was measured by the kinetic colorimetric Jaffe method (Cobas c702^®^), urea was measured by the kinetic urease and glutamate dehydrogenase methods (Cobas c702^®^). Serum total cholesterol and triglycerides was measured by the enzymatic colorimetric methods (Cobas c702^®^), and HDL (high density lipoprotein) cholesterol by the homogeneous direct method (Cobas c702A^®^), LDL (low density lipoprotein) cholesterol was calculated using the Friedewald formula. Blood count and platelets were measured by the light absorbance/impedance/flow cytometry methods (Sysmex XE 5000^®^).

A Welmy anthropometric balance, model R/1W-200 with a capacity of 200 kg and precision of 0.100 g was used to measure weight. A Tonelli stadiometer with a centimeter decimals scale was used to check heights. The blood pressure (BP) in both arms was measured with the patients in a seated position. After 10 min of rest it was done with a digital apparatus, Welch Ally, model Vital Signs Monitor 300 series.

The cutoff points adopted for BMI (body mass index) were those suggested by WHO (World Health Organization) [16], i.e. eutrophy (18.5–24.99); overweight (25–29.99) and obesity (≥ 30.00) [16]. The abdominal circumference (AC) was obtained at the midpoint between the last arch and the iliac crest and with a flexible and inelastic measuring tape without compressing the tissues. Abdominal obesity defined the abdominal circumference was greater than or equal 102 cm for men and greater than or equal to 88 cm for women. The interviewers were trained in the technique used to check the measurements of weight, height, and AC.

### Physical activity

Physical activity practiced by the individuals participating in the study was evaluated using the *International Physical Activity Questionnaire* (IPAQ), short version, classifying the level of physical activity as sedentary, irregularly active, active and very active, based on the information in meters/minute/week [17].

### Quality of life

Quality of life was evaluated using the WHOQOL BREF questionnaire version 1998 (WHO). For the purpose of evaluating quality of life, it is understood as a greater variety of conditions that may affect the individual’s perception, their feelings, and behavior related to their daily functioning, including but not limited to their health status and medical interventions [18].

### Chronotype and quality of sleep

The chronotypes and daily sleep preferences were investigated during the self-evaluation Munich Chronotype Questionnaire (MCTQ), elaborated and validated by Roenneberg et al. [19]. The MCTQ is a structured questionnaire on the behavior of wakefulness and sleep, and exposure to sunlight on work days and days of rest (day off). The interviewees inform how many days during the week they were involved in a regular work routine and the times at which they go to bed, are ready to go to sleep, fall asleep, and after how many minutes they get up, and how much time they spend outdoors. The following were calculated based on this information: sleep duration, midpoint of sleep for days of work and of rest, and social jetlag, which is the difference between the individual’s “biological clock”, and their “social clock”, that defines the social commitments of an individual throughout the day.

The diagnosis of Metabolic Syndrome was defined according to the updated criteria of the International Diabetes Federation (IDF) [20].

The criteria used to classify blood pressure was according to the 6th Brazilian Guidelines on Arterial Hypertension [21].

### Statistical analysis

Descriptive statistics were used to characterize the sample and the results were presented using central (median or mean) and variability measures (standard deviation or percentiles), as well as absolute and relative frequencies, according to the type of variable. Normality was verified using the Shapiro–Wilk test. The sociodemographic variables on health conditions, living habits, clinical and laboratory characteristics were presented and analyzed for all individuals. Statistical differences between the shifts and according to the sex of the professionals were evaluated using Pearson’s Chi Square tests (or Exact Fisher test, when necessary) and the Student t test (or Mann–Whitney) for the categorical and continuous variables, respectively.

The association of work shift with the outcomes of obesity (obese x normal plus overweight), excess weight (obese plus overweight x normal) and abdominal obesity (high vs low abdominal circumference) was evaluated through Logistic regression analyses, including hours of sleep, sleep duration during the week, social jetlag as predictors. The Wald test was used to test the significance of each variable in the model and the estimates of the odds ratio (OR) were presented with a 95% confidence interval. All factors were included in the multiple models, as well as a control for age and gender. The factor with the highest p-value was removed and this procedure was repeated until only significant factors were found or until only the effect of a factor to be controlled by age and sex remained.

The correlations of the MCTQ variables with the clinical and laboratory variables and age were evaluated through the Spearman correlation coefficient. Differences in the MCTQ score regarding the shift were evaluated using the Student t test (or Mann–Whitney) and the same analysis was performed stratifying it according to sex. As for BMI, ANOVA or Kruskal–Wallis, they were applied and were also stratified according to sex. Social jetlag was calculated based on the difference between the sleep on the work days and the free days, enabling a quantification of the difference between the biological and social clocks [19].

The quality of life scores were compared between the work shifts and between the number of hours of sleep, which was divided into two categories—more than 6 h and less or equal to 6 h—using the Student t test for 119 individuals. These comparisons were also performed with the stratified sample according to gender. All analyses were performed using the *Statistical Package for Social Sciences* (SPSS) software, version 21 and the statistical significance adopted was 5%.

## Results

Two hundred workers were included in the study, 69.5% (139) women and 30.5% (61) men. Considering the health conditions and living habits of the workers included in the sample, it was observed that the day shift workers had a higher prevalence of alcohol consumption (62.3%; p = 0.017), without a difference in the consumption profile (duration, quantity and type of beverage) between shifts. Night workers were older than day workers (Table 1).

**Table 1:** Sociodemographic, behavioral and occupational characteristics of workers according to work shift in southern Brazil (n: 200)

Variables	Total	Day (n = 94)	Night (n = 106)	p
Age (years) (n = 200)	43.2 ± 9.3	40.4 ± 9.2	45.6 ± 8.6	< 0.001*
Age < 50 years	140 (70)	75 (80)	65 (61.3)	0.008^##^
Age ≥ 50 years	60 (30)	19 (20)	41 (38.7)	
Gender (n = 200)				0.958^##^
Female	139 (69.5)	66 (70.2)	73 (68.9)	
Male	61 (30.5)	28 (29.8)	33 (31.1)	
Race (n = 200)				
White	155 (77.5)	74 (78.7)	81 (76.4)	0.825^##^
Non white	45 (22.5)	20 (21.3)	25 (23.6)	
Life partner (n = 200)				0.106^##^
Yes	89 (45.5)	48 (51.1)	41 (38.6)	
No	111 (55.5)	46 (48.9)	65 (61.4)	
Children (n = 124)				0.318^##^
Yes	92 (73.6)	39 (68.4)	53 (77.9)	
No	33 (26.4)	18 (31.6)	15 (22.1)	
Level of schooling (n = 200), years				0.668^#^
Up to the age of 8	11 (5.5)	5 (5.3)	6 (5.6)	
From 8 to 11	123 (61.5)	55 (58.5)	68 (64.2)	
Above 11	66 (33.0)	34 (36.2)	32 (30.2)	
Smoking (n = 117)				1.000^¥^
Smoker	11 (9.4)	5 (10.0)	6 (9.0)	
Non smoker	106 (90.6)	45 (90.0)	61 (91.0)	
Alcohol consumption (n = 118)				0.017^##^
Yes	58 (49.2)	33 (62.3)	25 (38.5)	
No	60 (50.8)	20 (37.7)	40 (61.5)	
Treatment for SAH		7 (12.5)	14 (20.6)	0.340^##^
Treatment for diabetes		2 (3.6)	6 (8.8)	0.292^¥^
Treatment for dyslipidemia		5 (8.9)	4 (5.9)	0.730^¥^
IPAQ (n = 112)				0.638^#^
Sedentary	14 (12.5)	5 (10.2)	9 (14.3)	
Irregularly active	14 (12.5)	7 (14.3)	7 (11.1)	
Active	61 (54.5)	29 (59.2)	32 (50.8)	
Very active	23 (20.5)	8 (16.3)	15 (23.8)	
Function (n = 200)				< 0.001^#^
Administrative	32 (16.1)	22 (23.7)	10 (9.4)	
Health professionals	125 (62.8)	44 (46.2)	82 (77.4)	
Support	32 (16.1)	23 (24.7)	9 (8.5)	
Maintenance	10 (5.0)	5 (5.4)	5 (4.7)	
Works elsewhere (n = 124)				0.340^#^
Yes	15 (12.1)	9 (16.1)	6 (8.8)	
No	109 (87.9)	47 (83.9)	62 (91.2)	
Income (n = 115), MW				0.001^#^
Up to 4	31 (27.0)	23 (44.2)	8 (12.7)	
From 4.1 to 5	46 (40.0)	14 (26.9)	32 (50.8)	
Above 5	38 (33.0)	15 (28.8)	23 (36.5)	
Time at hospital (years) (n = 200)	11.1 (4.8–17.5)	6.2 (2.5–12.5)	15.6 (9.3–20.0)	< 0.001**
Time on shift (years) (n = 200)	5.3 (1.4–13.8)	3.8 (1.3–11.9)	7.2 (1.6–15.7)	0.076**

Quantifiable variables with symmetrical distribution described by mean ± deviation. Quantitative variables with asymmetrical distribution described by the median (P25–P75). Categorical variables described by frequencies (percentages)

*MW* minimum wage

* Student test; ** Mann–Whitney test

^#^Pearson’s Chi Square test, ^##^ Pearson’s Chi Square test with continuity correction

^¥^Exact Fisher test

As for the classification of physical activity (IPAQ), only 6.7% of the male workers in the day shift were classified as very active, while in the night shift this prevalence was 35.3% (p = 0.034).

As for the WHOQOL brief, 60% (119) of the questionnaires were answered and 78 (65.5%) of the professionals evaluated their quality of life as being good. In the evaluation of domains, the physical domain presented the highest mean of scores (68.6 ± 15.9) followed by the social relations (66.6 ± 18.7), psychological (66.5 ± 14.1) and environmental domains (59.9 ± 14.5). Considering the work shifts, the day shift had better performance in all domains, except for the environment, but the differences were not significant.

The scores of the physical domain were lower in the sample that reported ≤ 6 h of sleep compared to those who slept > 6 h (65.0 ± 14.7 vs. 72.42 ± 16.9, respectively; p = 0.013). Similar behavior was observed for the psychological domain (63.6 ± 14.0 vs 70.1 ± 13.3, respectively; p = 0.013).

The night shift workers as a whole weighed more (77.9 ± 15.4 vs 72.4 ± 15.5; p = 0.011), BMI (28.7 ± 4.8 vs 26.4 ± 4.6; p < 0.001), and AC (96.4 ± 12.1 vs 89.9 ± 12.3; p 0.001) compared to the day shift workers. The men who worked at night had more abdominal obesity (100.7 ± 11.7 vs 92.9 ± 12.7, p-value = 0.017), and higher BMI (29.5 ± 4.3 vs 26.7 ± 4.7; p = 0.018) compared to the day shift. The women in the night shift presented similar results, with increased AC (92.9 ± 12.7 vs 88.7 ± 12.0; p = 0.009) and higher BMI compared to the day shift (28.4 ± 4.9 vs 26.3 ± 4.6; p = 0.014) (Table 2).

**Table 2:** Clinical and laboratory characteristics of workers between day and night shifts in Southern Brazil

Variables	General			Women			Men		
	Day	Night	p	Day	Night	p	Day	Night	p
Weight (kg) (n = 200)	72.4 ± 15.5	77.9 ± 15.4	0.011**	69 ± 13.1	72.6 ± 12.8	0.128**	80.5 ± 18.0	89.8 ± 14.2	0.030*
BMI (n = 200)	26.4 ± 4.6	28.7 ± 4.8	< 0.001**	26.3 ± 4.6	28.4 ± 4.9	0.014**	26.7 ± 4.7	29.5 ± 4.3	0.018*
AC (n = 200)	89.4 ± 12.3	96.4 ± 12.1	< 0.001**	88.7 ± 12.0	92.9 ± 12.7	0.009**	92.9 ± 12.7	100.7 ± 11.7	0.017*
Presence MS	19 (29.2)	32 (43.2)	0.125^##^	13 (28.9)	21 (40.4)	0.332^##^	6 (30.0)	11 (40.0)	0.315^##^
SAP-right (n = 200)	116.0 (109.3–127.8)	120.0 (110.0–131.0)	0.240**	116.0 (109.0–127.5)	120.0 (110.0–129.0)	0.591**	121.0 (111.0–128.0)	120.0 (112.5–138.0)	0.180**
SAP-left (n = 200)	117.0 (108.0–125.0)	119.0 (110.0–130.0)	0.196**	115.0 (108.0–123.0)	118.0 (109.0–127.0)	0.424**	119.0 (111.0–131.0)	125.0 (112.5–139.0)	0.223**
DAP right (n = 200)	75.5 (68.0–84.8)	78.0 (70.5–86.0)	0.269**	76.0 (68.0–85.5)	77.0 (70.0–84.0)	0.790**	75.7 ± 9.8	81.0 ± 11.2	0.060*
DAP left (n = 200)	76.4 ± 11.1	77.5 ± 10.5	0.458*	77.0 (68.0–84.8)	77.0 (69.5–83.0)	0.890**	75.6 ± 8.9	79.1 ± 12.0	0.205*
TC	192.7 ± 32.5	200.0 ± 36.0	0.225*	195.7 ± 33.4	200.5 ± 32.4	0.474*	185.2 ± 29.8	198.5 ± 44.9	0.294*
HDL	49 (44–61.5)	47 (40–55.3)	0.070**	55.3 ± 10.5	50.5 ± 12.2	0.041*	44.0 (36.8–48.5)	43.0 (39.8–47.5)	0.980**
LDL	121.0 ± 28.9	129.7 ± 35.0	0.155*	123.4 ± 30.7	127.6 ± 27.1	0.522*	116.9 ± 25.5	134.9 ± 50.2	0.183*
Triglycerides	97 (58.3–133.5)	100.0 (74.5–147.0)	0.257**	97.0 (58.0–125.0)	91.5 (68.5–135.4)	0.534**	100.0 (62.0–163.0)	120.0 (82.0–172.0)	0.294**
Glycemia	89.0 (81.0–95.8)	90.5 (85.0–99.8)	0.124**	89.0 (81.0–90.0)	89.0 (84.0–96.0)	0.215**	94.5 (80.8–99.0)	96.0 (88.0–108.0)	0.267**
HbA1c	5.4 (5.1–5.7)	5.5 (5.1–6.0)	0.271**	5.4 ± 0.5	5.5 ± 0.6	0.574	5.4 (5.2–5.5)	5.5 (5.1–6.6)	0.251**
Insulin	11.0 (8.5–15.8)	10.5 (7.6–13.9)	0.693**	11.1 (8.6–15.9)	12.2 (7.7–16.1)	0.789**	10.6 (7.5–15.2)	9.7 (7.5–10.7)	0.287**
Creatinine	0.7 (0.6–0.8)	0.7 (0.6–0.8)	0.487**	0.6 (0.5–0.7)	0.7 (0.6–0.7)	0.139**	0.9 (0.8–1.0)	0.9 (0.7–1.0)	0.657**
CKD-EPI GFR	111.0 (101.0–122.0)	104.5 (93.0–113.0)	0.017**	112.1 ± 14.4	103.7 ± 14.5	0.006*	102.8 ± 13.7	99.6 ± 19.5	0.583*
Urea	31.0 (26.0–37.0)	31.0 (27.0–38.0)	0.745**	29.0 (24.8–35.0)	30.0 (27.0–34.0)	0.429**	35.0 (29.5–41.0)	35.0 (30.0–41.0)	0.925**

Quantitative variables with symmetrical distribution described by mean ± deviation. Quantitative variables with asymmetrical distribution described by the median (P25–P75). Categorical variables described by frequencies and percentages. Lab tests information were available in 180 workers

*AC* abdominal circumference in cm, BMI body mass index, *CK EPI GFR* chronic kidney disease epidemiology collaboration estimate glomerular filtrate rate, *DAP* diastolic arterial pressure (mmHg), *HbA1c* glycated hemoglobin, *HDL* high density lipoprotein, *LDL* low density lipoprotein, *MS* metabolic syndrome, *SAP* systolic arterial pressure (mmHg), *TC* total cholesterol

* Student’s t test; ** Mann–Whitney test

^#^Pearson’ s Chi Square test; ^##^Pearson’s Chi Square test with continuity correction

The night shift workers presented lower glomerular filtration rates through CK-EPI [104.5 (93.0–113.0) vs 111.0 (101.0–122.0) p = 0.01]. Higher levels of HDL cholesterol were observed in female workers in the day shift compared to those in the night shift (55.3 ± 10.5 vs 50.5 ± 12.2; p-value = 0.041 (Table 2).

For night shift workers on their days off, they had a negative association between systolic BP (r_s_ = − 0.275; p = 0.040), diastolic BP (r_s_ = − 0.309; p = 0.019) with duration of sleep. A negative correlation was also observed between weight and social jetlag (r_s_ = − 0.301; p = 0.023) and between age and duration of sleep on work days (r_s_ = − 0.263; p = 0.048).

Analysis of the MCTQ parameters showed that the night shift workers had a midpoint of sleep on work days [5:30 (4:09–10:30) vs. 3:41 (2:48–4:31) p < 0.001], greater social jetlag [− 0:27 (− 6:26 to 0:06) vs. 1:03 (0:06–1:56) p < 0.001], and shorter duration of sleep on the work days (6:00 ± 2.27 vs. 7:03 ± 1.39 p 0.007) compared to the day shift workers (Table 3).

**Table 3:** Description of sleep variables according to gender and work shift, of workers in southern Brazil (n: 130)

MCTQ	General			Women			Men		
	Day	Night	p-value	Day	Night	p-value	Day	Night	p-value
Sleep midpoint working days (hours)	3:41 (2:48 to 4:31)	5:30 (4:09 to 10:30)	< 0.001**	3:45 (2:47 to 4:27)	4:45 (4:09 to 10:17)	< 0.001**	3:38 (2:47 to 4:38)	9:25 (5:07 to 12:38)	< 0.001**
Sleep duration working days (hours)	7:03 ± 1:39	6:00 ± 2:27	0.007*	7:11 ± 1:48	6.09 ± 2:39	0.040*	6:57 ± 1:20	5:50 ± 2:20	0.080*
Sunlight working days (hours)	1:00 (0:18 to 2:00)	1:00 (0:25 to 3:00)	0.433**	1:00 (0:30 to 2:00)	2:00 (0:40 to 3:00)	0.081**	2:00 (1:00 to 3:30)	1:00 (0:45 to 3:08)	0.262**
Sleep midpoint days off (hours)	4:47 (4:03 to 5:42)	4:30 (3:48 to 5:53)	0.772**	4:50 (3:40 to 6:00)	4:30 (4:00 to 5:40)	0.650**	4:40 (4:08 to 5:30)	4:57 (3:06 to 6:21)	0.783**
Sleep duration days off (hours)	8:10 ± 1:59	7:40 ± 2:12	0.046**	8:18 ± 2:21	7.54 ± 2:14	0.111**	8:01 ± 1:45	7:09 ± 2:18	0.191**
Sunlight days off (hours)	3:00 (2:00 to 5:00)	3:00 (1:09 to 4:09)	0.168**	2:45 (2:00 to 4:53)	2:30 (1:00 to 5:00)	0.741**	4:00 (3:00 to 6:30)	3:00 (1:11 to 4:00)	0.048**
Social jetlag	1:03 (0:06 to 1:56)	− 0:27 (− 6:26 to 0:06)	< 0.001**	1:00 (0:00− 2:17)	0:00 (− 5:47 to 0:12)	< 0.001**	1:15 (0:30− 1:45)	− 1:30 (− 7:50 to 0:05)	< 0.001**
Does not use alarm clock on days off	59 (85.5)	35 (62.5)	0.006^##^	40 (85.1)	26 (65.0)	0.053^##^	19 (86.4)	9 (56.3)	0.088^##^
Awakes with the alarm clock	26 (52.0)	7 (24.1)	0.029^##^	18 (54.5)	4 (20.0)	0.029^##^	8 (47.1)	3 (33.3)	0.797^##^

* Student t test; ** Mann–Whitney test

^##^Pearson’s Chi Square test with continuity

In the analysis without adjustment, the odds of abdominal obesity were 2.5 times higher in the night shift workers than the day shift ones (OR = 2.53 (CI 95% 1.43–4.50); p = 0.002). The shift remained significantly associated with abdominal obesity after controlling for age and gender, where the odds were almost 3 times higher [OR = 2.93 (CI 95% 1.57–5.46); p < 0.001] for the employees who work at night compared to the day shift workers (Table 4). Adjustments for jetlag and hours sleep duration were not significant.

**Table 4:** Individual models of raw and adjusted regressions for outcomes of abdominal obesity, excess weight and obesity of workers in southern Brazil, (n: 200)

	Variables	n (%)	OR_raw (_CI 95%)	p-value	OR_adjusted (_CI 95%)^#^	p-value
Abdominal obesity	Social jetlag	–	0.93 (0.84–1.03)	0.141	0.96 (0.85–1.08)	0.485
	Hours of sleep	–	0.86 (0.71–1.04)	0.128	1.05 (0.73–1.52)	0.779
	Sleep duration WD		0.98 (0.83–1.15)	0.778	1.08 (0.88–1.33)	0.462
	Shift day	39 (42.4)	1		1	
	Shift night	69 (65.1)	2.53 (1.43–4.5)	0.002	2.93 (1.57–5.46)	0.001
Excess weight	Social jetlag	–	0.86 (0.76–0.98)	0.026	0.87 (0.76–0.99)	0.040
	Hours of sleep	–	0.75 (0.60–0.94)	0.014	1.03 (0.69–1.54)	0.875
	Sleep duration WD		0.87 (0.72–1.04)	0.129	0.94 (0.76–1.16)	0.563
	Shift day	53 (57.6)	1		1	
	Shift night	84 (79.2)	2.81 (1.50–5.25)	0.001	1.69 (0.70–4.04)	0.241
Obesity	Social jetlag	–	0.88 (0.80–0.98)	0.017	0.88 (0.79–0.98)	0.020
	Hours of sleep	–	0.89 (0.73–1.09)	0.260	0.92 (0.68–1.25)	0.583
	Sleep duration WD		0.93 (0.78–1.11)	0.413	1.09 (0.78–1.53)	0.619
	Shift day	20 (21.7)	1		1	
	Shift night	41 (38.7)	2.27 (1.20–4.26)	0.011	1.05 (0.29–3.81)	0.940

*WD* work day

^#^Adjustment for sex and age (≥ 50 vs < 50)

The night shift workers presented 2.8 times higher odds of developing excess weight than the day shift [OR = 2.81 (CI 95% 1.50–5.25); p = 0.001] and for every increased unit of social jetlag and hour of sleep there was 14% [OR = 0.86 (CI 95% 0.76–0.98); p = 0.026] and 25% [OR = 0.75 (CI 95% 0.60–0.94); p = 0.014] smaller odds of presenting excess weight, respectively. In the multiple regression analysis, the odds of excess weight were reduced by around 13% (OR = 0.87 (CI 95% 0.76–0.99); p = 0.040) for every increased unit of social jetlag (Table 4).

The odds of developing obesity were 2.3 times higher in night shift workers compared to the day shift [OR = 2.27 (CI 95% 1.20–4.26); p = 0.011] and for every increased unit of social jetlag there was 12% (OR = 0.88 (IC 95% 0.80–0.98); p = 0.017) of reduction. In the multiple regression analysis, only the protection effect of jetlag was maintained (OR = 0.88 (IC 95% 0.79–0.98); p = 0.020) (Table 4).

Among the night shift workers, a higher prevalence of people who sleep a smaller amount of hours (equal to or less than 6 h/day) was observed, compared to day shift workers (p = 0.003). In the stratified analysis for gender, it was observed that this behavior was present only in women night shift workers compared to the day shift ones, 66% vs. 33% (p = 0.006) respectively.

## Discussion

This study evaluated the association between night work and obesity. The results showed that night work was a determining risk factor for abdominal obesity. Social jetlag was higher in night shift workers and it was also associated with obesity.

The night shift workers had less hours of sleep compared to people who worked in the daytime. They also presented more obesity, overweight and higher AC. Night shift workers also had worse associations between BP and sleep. As well, in the MCTQ questionnaire they have higher social jetlag and lower sleep. After adjustments, the social jetlag was an important predictor of obesity.

The night shift workers, even though sleeping more on their days off, did not regain the sleep deficit produced by the working days. Sleep debt may be due to many reasons, mainly the presence of light during the time for sleeping, the increased temperature and an altered cortisol secretion [22]. Sleeping more than 6 h was associated with better performance in the physical and psychological domains, reinforcing the importance of good sleep quality.

In this sample, the male night shift workers were more active than day shift workers. This can be explained because in our hospital there is a gymnasium for the employees, facilitating workers’ access to regular physical exercise. Studies that evaluated these aspects among night shift workers are limited and with diverse results, possibly related to the study population, the methodology used and the definitions of night work [23, 24]. In our data, the same result for female night shift workers was not found, we believe that women have a second shift of work at home, taking care of their family.

In our study, there was no relationship between work shift and laboratory variables, although other studies demonstrate a higher prevalence of metabolic disorders in these workers [24, 25]. The effects of night work on these variables can be difficult to demonstrate in a cross-sectional study.

The association between night work or shift work and increased body weight is present in some studies [25, 26], however, the pathophysiological mechanisms of this association are not yet clear. A systematic review performed by Van Drongelen [27] found an association between shift work and increased body weight, and the generalization of these findings is limited by the heterogeneity of the studies included in the meta-analysis.

A Brazilian study performed at 18 public hospitals with 2371 nursing staff [28] identified an association between years of exposure to night work and BMI for both genders after adjusting for all covariables. The effect of night work was greater among the men than among the women.

A systematic review with a meta-analyze evaluated 28 studies and observed that night work increased the risk of obesity/overweight by 23% and the risk for abdominal obesity [29] by 35%, considering the years of exposure and number of shifts per month.

Leproult et al. concluded that the risk of obesity, in general, increases for individuals who sleep less than 6 h, with a significant association between short duration of sleep and weight gain, incidence of overweight and obesity throughout the follow up period [13]. More recently, an American study with 883 individuals [30] demonstrated that the worst quality and shortest duration of sleep were significantly associated with the incidence of obesity (OR: 1.10; CI 95% 1.03–1.18).

The desynchronization of rhythmic physiological functions, that also impairs the duration and quality of sleep, interferes in the control of food intake, manifested metabolically by the increased levels of ghrelin and reduced levels of leptin, and may contribute to possible pathological mechanisms [31].

Among the possible limitations of our study there is emphasis on a cross-sectional design which does not allow establishing the causal relationship between shift work and obesity, and the absence of an evaluation of the food profile of the cohort studied, which might help differentiate shifts regarding eating behavior. The healthy worker effect is another aspect to be considered in relation to the selection of the sample. However, such factors do not compromise the results, given the scarcity of information and the relevance of the topic discussed.

In order to support the evidence, cohort studies are needed to elucidate the contribution of night work and sleep debt to overweight/obesity and even to cardiovascular outcomes.

## Data Availability

The datasets generated and/or analyzed during the current study are not publicly available due [reason why the data is not public] but are available from the corresponding author on reasonable request.

## Abbreviations

AC: Abdominal circumference
BP: Blood pressure
BMI: Body mass index
CK EPI GFR: Estimate glomerular filtrate rate
HDL: High density lipoprotein
IDF: International Diabetes Federation
IPAQ: International Physical Activity Questionnaire
LDL: Low density lipoprotein
MCTQ: Munich Chronotype Questionnaire
OR: Odds ratio
SAH: Systemic arterial hypertension
SPSS: Statistical Package for Social Sciences
WHO: World Health Organization
